# Effects of Fatty Acid Anhydride on the Structure and Thermal Properties of Cellulose-g-Polyoxyethylene (2) Hexadecyl Ether

**DOI:** 10.3390/polym10050498

**Published:** 2018-05-04

**Authors:** Wanyong Yu, Na Han, Yongqiang Qian, Xingxiang Zhang, Wei Li

**Affiliations:** Tianjin Municipal Key Laboratory of Advanced Fiber and Energy Storage, School of Materials Science and Engineering, Tianjin Polytechnic University, Tianjin 300387, China; yu20130513@163.com (W.Y.); qianyongqiang100@sina.com (Y.Q.); hiweilee@gmail.com (W.L.)

**Keywords:** cellulose, fatty acid anhydride, polyoxyethylene (2) hexadecyl ether, solid–solid phase change

## Abstract

Cellulose was premodified by short-chain fatty acid anhydrides, such as acetic anhydride (CA), propionic anhydride (CP), and butyric anhydride (CB), followed by grafting of polyoxyethylene (2) hexadecyl ether (E_2_C_16_) using toluene-2,4-diisocyanate as a coupling agent. The feeding molar ratio of E_2_C_16_ and the anhydroglucose unit (AGU) was fixed at 4:1, and then a series of CA-g-E_2_C_16_, CP-g-E_2_C_16_, and CB-g-E_2_C_16_ copolymers were successfully prepared. The structures and properties of the copolymers were characterized using FTIR (fourier transform infrared spectra), ^1^H-NMR (Proton nuclear magnetic resonance), DSC (Differential scanning calorimeter), POM (polarized light microscopy), TGA (thermogravimetric analysis) and WAXD (wide-angle X-ray diffraction). It was shown that with the anhydride/AGU ratio increasing, the degree of substitution (DS) value of E_2_C_16_ showed a trend of up first and then down. With the carbon chain length increasing, the DS value of E_2_C_16_ continuously increases. The phase transition temperature and thermal enthalpy of the copolymers increased with an increasing DS value of E_2_C_16_. When the ratio of CB/AGU was 1.5:1, the DS of E_2_C_16_ was up to the maximum value of 1.02, and the corresponding melting enthalpy and crystallization enthalpy were 32 J/g and 30 J/g, respectively. The copolymers showed solid–solid phase change behavior. The heat resistant temperature of cellulose-based solid–solid phase change materials was always higher than 270 °C. After the grafting reaction, the crystallinity of E_2_C_16_ decreased, while the crystal type was still hexagonal.

## 1. Introduction

Cellulose is a type of polysaccharide composed of repetitive d-glucose units linked through β(1→4) glycosidic bonds [1]. There are three active –OH groups in each d-glucose unit, and these can easily form strong intramolecular and intermolecular hydrogen bonds, resulting in smaller distances between molecules and rigid chains. As a result, cellulose is insoluble in common organic solvents and unable to be melt-processed before reaching its decomposition temperature [2,3]. Through modification [4,5,6,7,8,9], hydrogen bonding was weakened to a certain extent, while a grafted side chain increased molecular distance, which could further improve the solubility of cellulose. Furthermore, the increased molecular distance is helpful for the grafting reaction [10]. When the degree of substitution reaches a critical point, the cellulose derivatives will be melt-processable.

Vaca-Garcia et al. [11] found that a grafted side chain could weaken the interaction between the molecular chains of cellulose, so that cellulose esters soften and flow at comparatively low temperatures. However, if the side chain is too long (C > 6), the long molecular chains account for most of the free volume, resulting in motion of the cellulose ester backbone becoming difficult. That is to say, a flexible long-chain has lost the advantage of lowering the melting point of the cellulose ester. However, if a bulky group (such as benzene) was introduced into the cellulose back-bone, there will be more free volume, and as a result, the thermal flow temperature of cellulose ester will be depressed.

Xiao et al. [12] prepared cellulose esters containing phosphorus, cellulose diphenyl phosphate (C-Dp), and cellulose acetate (CA)–diphenyl phosphate mixed esters (C-A–Dp). C-A–Dp showed clear glass transition behavior. All the products possessed excellent solubility in common organic solvents (e.g., CHCl_3_, acetone, ethyl acetate), and transparent films of cellulose esters were obtained by solution casting. Furthermore, these cellulose mixed esters exhibited thermoplastic behavior and could be processed by a traditional melt processing method. Luan et al. [13] conducted cellulose esterification in a homogeneous system, where acetic anhydride (CA) and 1-allyl-3-methylimidazolium chloride (AmimCl) were used as the grafting comonomer and solvent, respectively, followed by the ring-opening grafting of l-lactide. As a result, a series of cellulose acetate-g-polylactic acid (CA-g-PLA) copolymers were obtained. The CA-g-PLA copolymers had glass transition temperatures (T_g_) in the range of 60–130 °C and exhibited thermoplastic behavior.

In this study, fatty acid anhydride, CA, propionic anhydride (CP), and butyric anhydride (CB) were selected as esterification agents for cellulose, followed by the grafting of polyoxyethylene (2) hexadecyl ether (E_2_C_16_) using toluene-2,4-diisocyanate as a coupling agent and the feeding molar ratio of E_2_C_16_/AGU fixed at 4:1, and then CA-g-E_2_C_16_, CP-g-E_2_C_16_, and CB-g-E_2_C_16_ copolymers were obtained. The effect of carbon chain length and usage amount of fatty acid anhydrides on structures and properties of cellulose-based solid–solid phase change materials were investigated in detail.

## 2. Experimental

### 2.1. Materials

Microcrystalline cellulose (MC, DP (Degree of polymerization) = 220) was purchased from Beijing Fengli Jingqiu Commerce and Trade Co. (Beijing, China); AmimCl was purchased from Shanghai Chengjie Chemical Co. (Shanghai, China); *N*,*N*-Dimethylformamide (DMF), dibutyltin dilaurate (DBTDL), acetic anhydride (AA), and pyridine were purchased from Tianjin Guangfu Technology Development Co. (Tianjin, China); propionic anhydride (PA) was purchased from TCI Chemical Reagent Co. (Shanghai, China); butyric anhydride (BA) was purchased from Aladdin Chemical Reagent Co. (Shanghai, China); Toluene 2,4-diisocyanate (TDI) was purchased from Tianjin Fuchen Chemical Reagent Factory. (Tianjin, China); E_2_C_16_ was purchased from Aldrich Chemical Reagent Co. (Shanghai, China); All reagents were used without further purification.

### 2.2. Cellulose Esterification

MC was dissolved completely in AmimCl at 80 °C. Then, the desired amounts of fatty acid anhydrides and pyridine were added into cellulose solution. The esterification was conducted for 2 h at 90 °C. 

### 2.3. Preparation of Prepolymer

The desired amounts of TDI and E_2_C_16_ (molar ratio 1.1:1) were dissolved in DMF separately. E_2_C_16_ solution was added dropwise into the TDI solution with continuous stirring at 25 °C for 15 min, and then the reaction continued for 60 min. The prepolymer solution was obtained when the reaction finished.

### 2.4. Preparation of Cellulose-Based Copolymers

The prepolymer solution and DBTDL (0.1 wt % of TDI) were added to cellulose ester solution. The mixture was stirred at 90 °C for 6 h. When the grafting reaction was complete, the reaction mixture was precipitated in distilled water for 12 h. Then, the crude product was filtered and soaked in acetone for another 48 h to dissolve ungrafted prepolymer in precipitation. After that, the product was filtered, dried, and weighed.

**Scheme 1:**
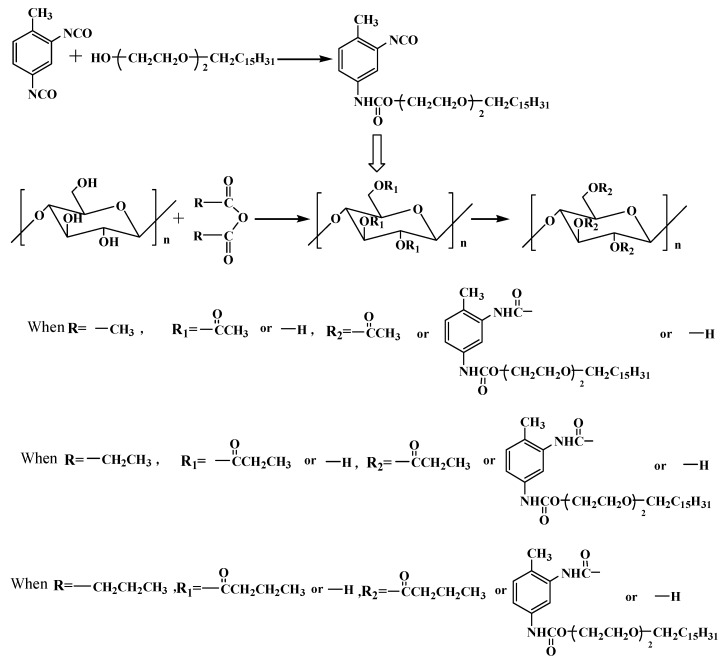
Synthesis mechanism of CA-g-E_2_C_16_, CP-g-E_2_C_16_, and CB-g-E_2_C_16_ copolymers.

The synthesis procedure of CA-g-E_2_C_16_, CP-g-E_2_C_16_, and CB-g-E_2_C_16_ copolymers are shown in Scheme 1, sample numbers and raw material feeding ratios are listed in Table 1.

### 2.5. Characterization

#### 2.5.1. FTIR

Fourier transform infrared spectra (FTIR) were collected using a Bruker Tensor-37 Fourier (Germany) transform infrared spectrometer. The dried samples were tested by the KBr disc technique, and infrared spectra were recorded in the range of 4000–400 cm^−1^.

#### 2.5.2. ^1^H-NMR

Proton nuclear magnetic resonance (^1^H-NMR) spectroscopy was performed using an Avance AV-300 MHz nuclear magnetic resonance spectrometer. The solvent was dimethyl sulfoxide-*d*_6_.

The degree of substitution (DS) of fatty acid group and E_2_C_16_ could be calculated by ^1^H-NMR results. 

*DS*_1_ of an acetyl group could be calculated by Equation (1) as follows:

(1) $$DS_{1}=\frac{7}{3}\cdot \frac{I_{CH3}}{I_{AGU}}$$

where *I_AGU_* is the integral intensity of the hydrogen atoms in the AGU and C_6_ (7H, σ = 3.0~5.5) and *I_CH_*_3_ is the integral intensity of the –CH_3_ group (3H, δ = 1.7~2.2) in an acetyl group.

*DS*_1_ of a propionyl group could be also calculated by Equation (1), where *I_CH_*_3_ is the integral intensity of the –CH_3_ group (3H, δ = 1.1) in a propionyl group.

*DS*_1_ of a butyryl group could be calculated by Equation (2) as follows:

(2) $$DS_{1}=\frac{7}{2}\cdot \frac{I_{CH2}}{I_{AGU}}$$

where *I_CH_*_2_ is the integral intensity of the –CH_2_– group (2H, δ = 1.6) in a butyryl group.

*DS*_2_ of E_2_C_16_ could be calculated by Equation (3) as follows:

(3) $$DS_{2}=\frac{7}{3}\cdot \frac{I_{phenyl}}{I_{AGU}}$$

where *I_phenyl_* is the integral intensity of benzene (3H, δ = 7.0~7.4) in TDI.

#### 2.5.3. DSC

Differential scanning calorimetry (DSC) was performed using a NETZSCH DSC 200 F3 (Germany). The samples were heated from 20 °C to 250 °C at 30 °C/min to eliminate the thermal history. Then, the samples were cooled to −20 °C and heated from −20 °C to 250 °C at 10 °C/min. All heating and cooling processes were performed under a nitrogen atmosphere. The thermal data were read from the second heating run and the first cooling run.

#### 2.5.4. POM

The type of phase change was carried out with hot-stage polarized light microscopy using a BX51TRF. Samples were firstly heated to 100 °C and then cooled down to room temperature (20 °C) in order to remove the thermal history. Subsequently, they were heated to 100 °C at a heating rate of 10 °C/min to observe the thermal behaviors.

#### 2.5.5. TG

Thermogravimetric analysis (TGA) was carried out in a NETZSCH STA 449F3 from 40 °C to 800 °C at a heating rate of 10 °C/min under a nitrogen atmosphere.

#### 2.5.6. WAXD

Wide-angle X-ray diffraction (WAXD) was recorded on a D/MAX-2500 under conditions of 150 mA and 40 kV at 0 °C. The X-ray source is an 18-kW rotating anode X-ray generator equipped with a Cu target. The incident X-ray beam was monochromated by a pyrolytic graphite, and the data were measured in a range of 2θ = 4°~40°, λ = 1.54 Å.

## 3. Results and Discussion

### 3.1. FTIR

**Figure 1:**
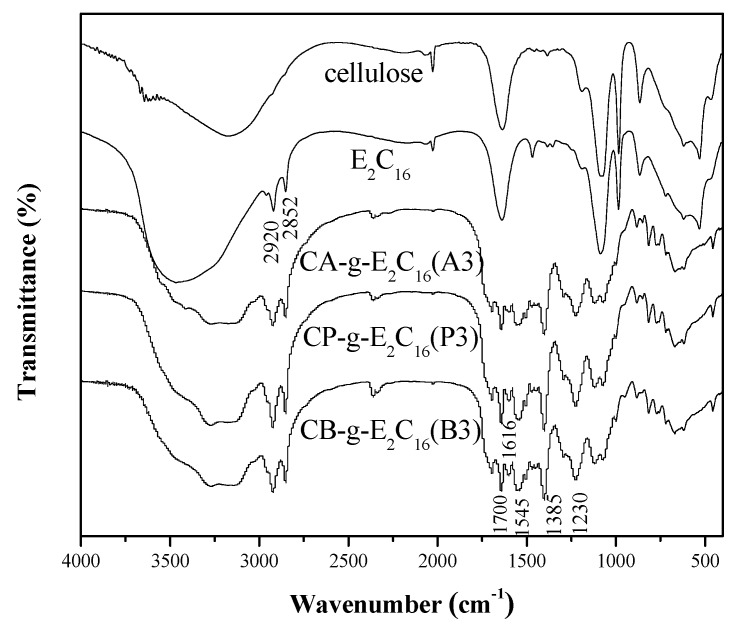
FTIR spectra of cellulose, E_2_C_16_, CA-g-E_2_C_16_ (A3), CP-g-E_2_C_16_ (P3), and CB-g-E_2_C_16_ (B3) copolymers.

Figure 1 displays the FTIR spectra of cellulose, E_2_C_16_, CA-g-E_2_C_16_ (A3), CP-g-E_2_C_16_ (P3), and CB-g-E_2_C_16_ (B3) copolymers. In the spectrum of cellulose, the wide peak at approximately 3200 cm^−^^1^ is assigned to the absorption band of the hydroxyl groups on the AGU, whose intensity was significantly reduced in the spectra of copolymers. In the spectrum of E_2_C_16_, the peaks at 2852 cm^−1^ and 2920 cm^−1^ are assigned to the characteristic absorption bands of the ethylene glycol repeat units and the saturated alkane group of E_2_C_16_, respectively, which also appeared in the spectra of CA-g-E_2_C_16_ (A3), CP-g-E_2_C_16_ (P3), and CB-g-E_2_C_16_ (B3) copolymers. In the spectrum of copolymers, 1385 cm^−1^ is assigned to the absorption band of C–H, 1230 cm^−1^ is assigned to the absorption band of C–O, and 1750 cm^−1^ is assigned to the absorption band of C=O. It is demonstrated that fatty acid groups (acetyl group, propionyl group, and butyryl group) were successfully linked onto the cellulose backbone. We can observe the characteristic absorption band of the conjugated C=C double bond of the benzene ring in TDI at 1603 cm^−1^ and the amide bands [14] at 1700 cm^−1^ and 1545 cm^−1^ in the spectra of copolymers, which indicated that the E_2_C_16_ was successfully grafted onto the cellulose backbone through TDI. Based on the analysis above, it could be declared that CA-g-E_2_C_16_, CP-g-E_2_C_16_, and CB-g-E_2_C_16_ copolymers were successfully synthesized.

### 3.2. ^1^H-NMR

**Figure 2:**
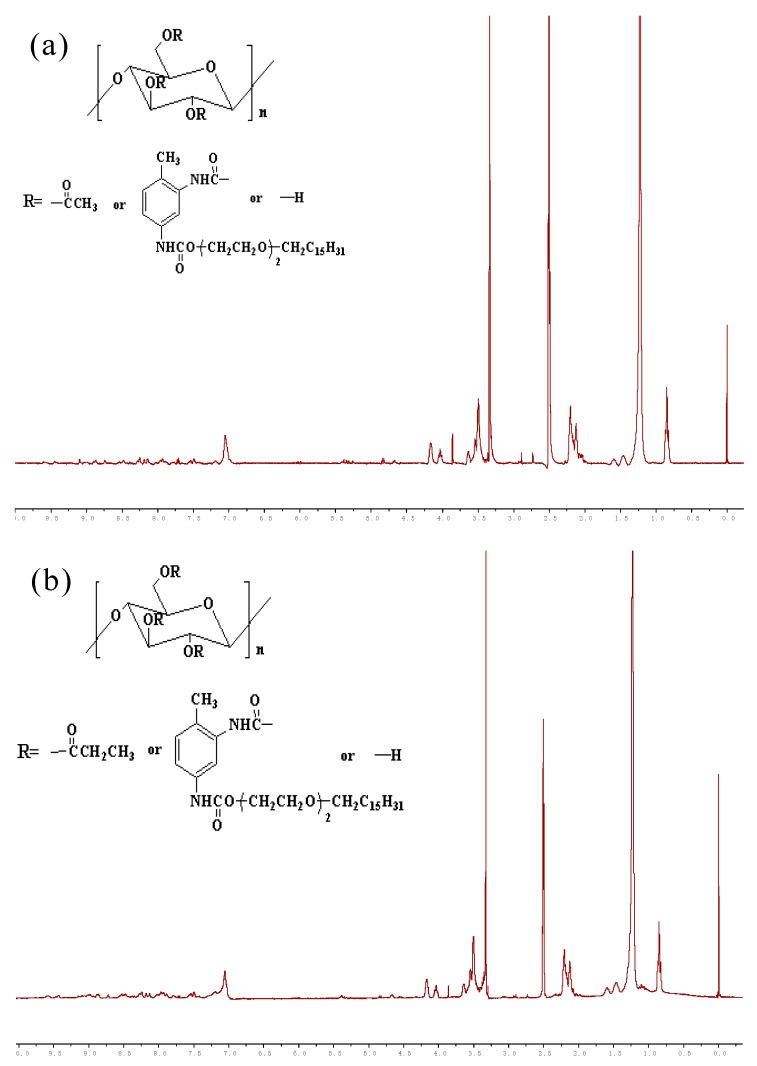

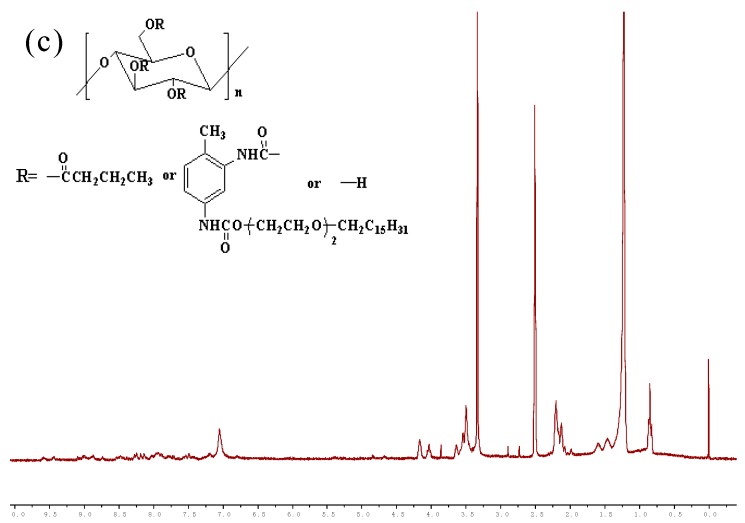
^1^H-NMR spectra of (**a**) CA-g-E_2_C_16_ (A3), (**b**) CP-g-E_2_C_16_ (P3), and (**c**) CB-g-E_2_C_16_ (B3) copolymers.

Figure 2 shows the ^1^H-NMR spectrum of CA-g-E_2_C_16_ (sample A3, a), CP-g-E_2_C_16_ (sample P3, b), and CB-g-E_2_C_16_ (sample B3, c) copolymers. The peaks at δ = 1.7~2.2 were assigned to the signals of the methyl protons of acetyl group, the peak at δ = 1.1 was assigned to the signal of the methyl protons of propionyl group, the peak at δ = 2.4 was assigned to the signal of the methylene protons of propionyl group, the peak at δ = 0.9 was assigned to the signal of the methyl protons of butyryl group, the peaks at δ = 1.6 and δ = 2.3 were assigned to the signals of the methylene protons of butyryl group [15], and the peaks at σ = 3.0~5.5 were assigned to the signals of the protons in the glucose unit ring and C6. It is also demonstrated that fatty acid groups were successfully linked onto the cellulose backbone. The peak at σ = 0.85 was assigned to the signal of the methyl protons of the alkyl chain in E_2_C_16_, the peaks at σ = 1.1~1.7 were assigned to the signals of the methylene protons of the alkyl chain in E_2_C_16_, the peak at σ = 3.4 was assigned to the signal of the methylene protons adjacent to the ether bond of the alkyl chain in E_2_C_16_, the peaks at σ = 3.5~3.8 were assigned to the signals of the protons of the ethylene glycol repeat units of E_2_C_16_, and the peaks at σ = 7.0~7.4 were assigned to the signals of the protons in the benzene ring of TDI. Based on the analysis above, it is evident that CA-g-E_2_C_16_, CP-g-E_2_C_16_, and CB-g-E_2_C_16_ copolymers were successfully synthesized.

The DS values of CA-g-E_2_C_16_, CP-g-E_2_C_16_, and CB-g-E_2_C_16_ copolymers are shown in Table 2. When the ratio of acid anhydride/AGU increased from 0.5:1 to 1.5:1, both *DS*_1_ and *DS*_2_ increased significantly. When the ratio of acid anhydride/AGU increased to 2:1, *DS*_1_ continued to increase while *DS*_2_ dropped. When the ratio of acid anhydride/AGU increased within a certain range, *DS*_1_ and the distance between cellulose molecules increased due to the anhydride side group. This is helpful for the grafting reaction of E_2_C_16_, so *DS*_2_ increased, as expected. However, when the feeding ratio of anhydride/AGU was higher than 1.5 mol %, *DS*_1_ continued to increase, the number of free hydroxyl groups in AGU decreased, and the intermolecular steric hindrance became stronger, leading to a lower *DS*_2_ value.

The calculated results showed that *DS*_2_ becomes larger with increasing carbon atom number of acid anhydride because the distance between cellulose molecules increases and the free volume region increases. However, the *DS*_1_ value of cellulose-based copolymers were roughly equal. This is because with the carbon atom number increasing, the effect of increasing distance between cellulose molecules becomes stronger, which is helpful for the grafting reaction of E_2_C_16_. We can conclude that the effect of increasing cellulose molecule spacing is in the order of BC > PC > AC. When the ratio of BC/AGU was 1.5:1, *DS*_2_ of E_2_C_16_ in all three types of copolymers reached the maximum value of 1.02.

### 3.3. DSC

**Figure 3:**
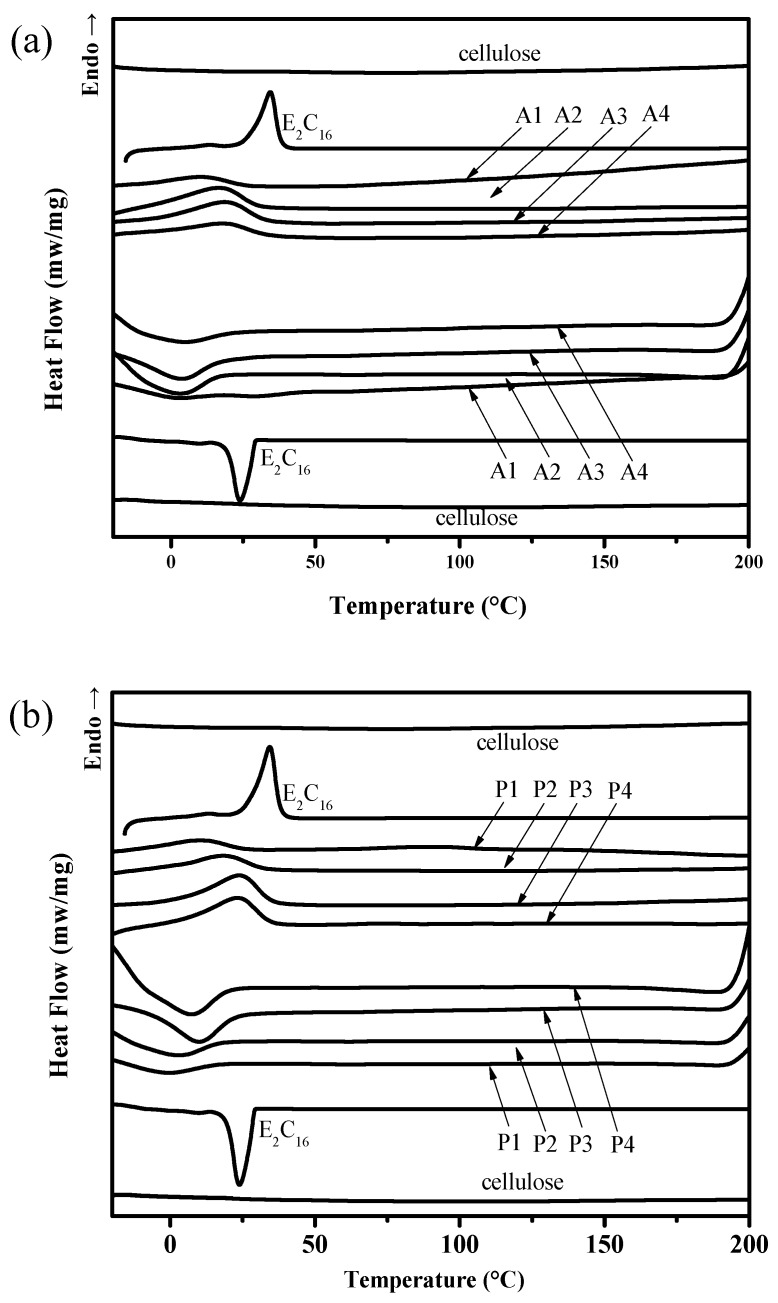

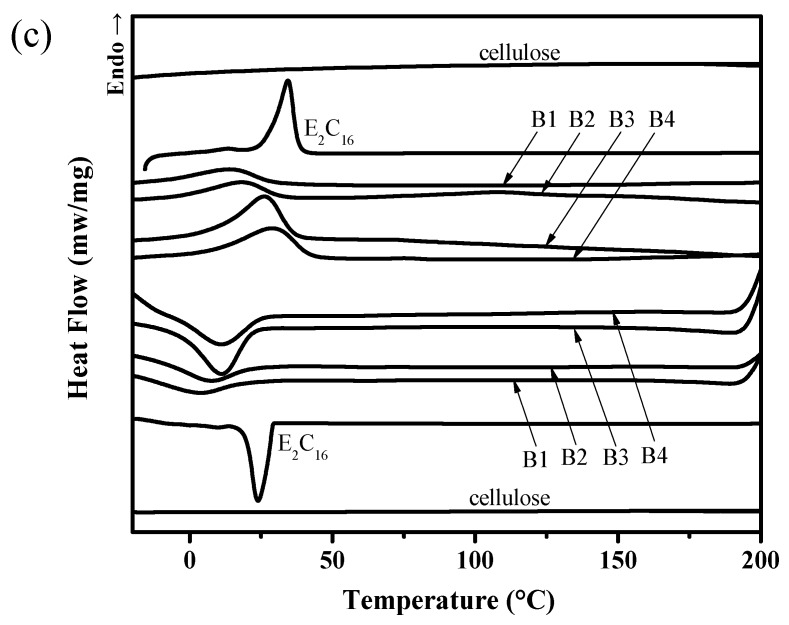
DSC curves of cellulose, E_2_C_16_, (**a**) CA-g-E_2_C_16_; (**b**) CP-g-E_2_C_16_, and (**c**) CB-g-E_2_C_16_ copolymers.

**Figure 4:**
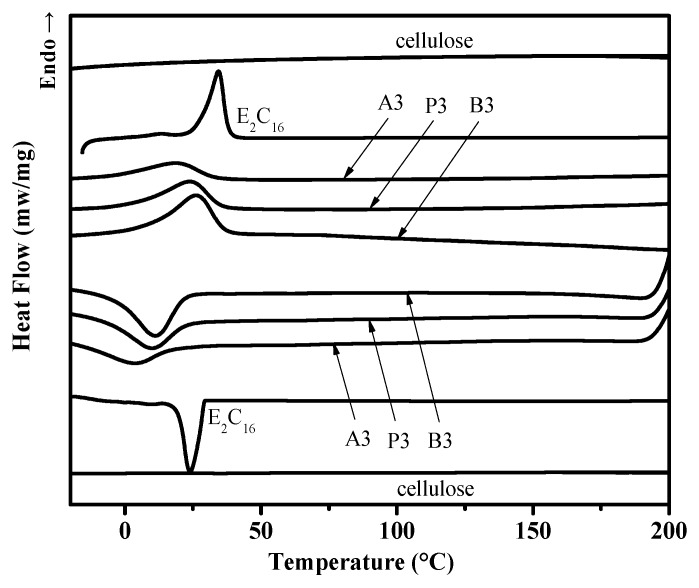
DSC curves of cellulose, E_2_C_16_, cellulose-g-E_2_C_16_ (S-B), CA-g-E_2_C_16_ (A3), CP-g-E_2_C_16_ (P3), and CB-g-E_2_C_16_ (B3) copolymers.

Figure 3 and Figure 4 and Table 3 show the thermal transition behavior of E_2_C_16_, CA-g-E_2_C_16_, CP-g-E_2_C_16_, and CB-g-E_2_C_16_ copolymers with different DS values. Pure E_2_C_16_ possessed distinct phase transition peaks with large latent heats (ΔH_m_ = 104 J/g; ΔH_c_ = 95 J/g), proving it is a type of phase-change material with good thermal storage capacity. For CA-g-E_2_C_16_, CP-g-E_2_C_16_, and CB-g-E_2_C_16_ copolymers, E_2_C_16_ is the working phase change material (PCM), while the cellulosic backbone acts as the supporting skeleton for fixing the E_2_C_16_ chains and contributes nothing to the enthalpy. Therefore, the thermal behavior of cellulose-based copolymers is in line with the changing trend of DS, as mentioned above.

The enthalpy is a linear relationship with the crystalline d-spacing [10,16]. As shown in Table 3, when the feeding ratio of fatty acid anhydride/AGU increased from 0.5:1 to 2.0:1, the value of *DS*_1_ continuously increased, while the *DS*_2_ reached a maximum value at 1.5:1 of fatty acid anhydride/AGU. It is ascribed to the steric hindrance effect derived from fatty acid anhydride. The reactivity of free radical polymerization is strongly affected by the steric hindrance of side chains, which rises with increase of the DS of side chains. Therefore, with an increasing fatty acid anhydride/AGU ratio, phase change enthalpy (ΔH), T_mo_ and T_do_ of the copolymers all exhibited a similar trend of first increasing and then decreasing, and they also reached a maximum value at 1.5:1 of fatty acid anhydride/AGU. Similarly, when the acid anhydride content was the same, the phase change enthalpy of the copolymers also increased with increasing the number of fatty acid anhydride carbon atoms, which is in line with the changing trend of *DS*_2_. For the CB-g-E_2_C_16_ copolymer, when the feeding ratio of BC/AGU was 1.5:1, *DS*_2_ in all copolymers reached the maximum value of 1.02, while ΔH_m_ and ΔH_c_ also showed the maximum values of 32 J/g and 30 J/g, respectively. In addition, compared with pure E_2_C_16_, the phase transition peaks of the copolymers shifted slightly to a lower temperature due to the increase in the side-chain crystal imperfection.

As shown in Figure 4, with increasing number of fatty acid anhydride carbon atoms, T_m_ and T_c_ of the copolymers follow the order CA-g-E_2_C_16_ < CP-g-E_2_C_16_ < CB-g-E_2_C_16_. Furthermore, the peak area follows the same order. This means that increasing the length of the carbon chain helps in improving the thermal storage capability.

Compared with ΔH_m_* based on E_2_C_16_ content in the blends, ΔH_m_ based on E_2_C_16_ content in the graft copolymers had a higher value. The movement of grafted E_2_C_16_ chains is confined so that they cannot freely arrange to crystallize, causing the imperfection of the E_2_C_16_ crystals to increase and resulting in a decrease in enthalpy. This also demonstrates that the products were cellulose-based copolymers. 

### 3.4. POM

**Figure 5:**
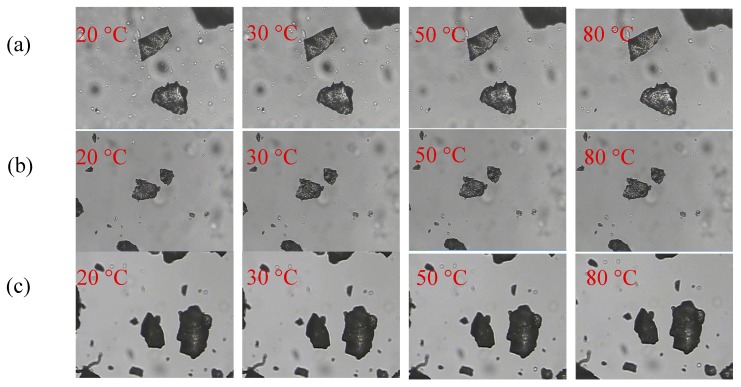
Polarized light microscopy (POM) photos of (**a**) CA-g-E_2_C_16_ (A3), (**b**) CP-g-E_2_C_16_ (P3), and (**c**) CB-g-E_2_C_16_ (B3) copolymers at different temperatures.

Figure 5 shows POM photos of CA-g-E_2_C_16_ (A3), CP-g-E_2_C_16_ (P3), and CB-g-E_2_C_16_ (B3) copolymers heated in the temperature range of 20~80 °C. As listed in Table 3, the melting onset temperatures of A3, P3, and B3 are 8.0 °C, 13.7 °C, and 14.2 °C, respectively. For CA-g-E_2_C_16_ (A3), when the environmental temperature rose from 20 °C to 50 °C, the E_2_C_16_ side chain of the copolymer experienced melting, and its phase changed from crystalline to amorphous morphology. However, its macroscopic state did not change from the solid state. When the temperature continued to rise to 80 °C, the macroscopic state of the copolymer still was not changed. This is because the melted E_2_C_16_ side chain is confined by the cellulosic backbone, and it cannot flow freely; therefore, the CA-g-E_2_C_16_ copolymer stayed in a solid state, on the whole. CP-g-E_2_C_16_ (P3) and CB-g-E_2_C_16_ (B3) copolymers also showed the same phenomenon. This demonstrated that the phase change type of the synthesized copolymers is solid–solid phase change; that is to say, CA-g-E_2_C_16_, CP-g-E_2_C_16_, and CB-g-E_2_C_16_ copolymers are a type of solid–solid phase material (SSPCM).

### 3.5. TGA

**Figure 6:**
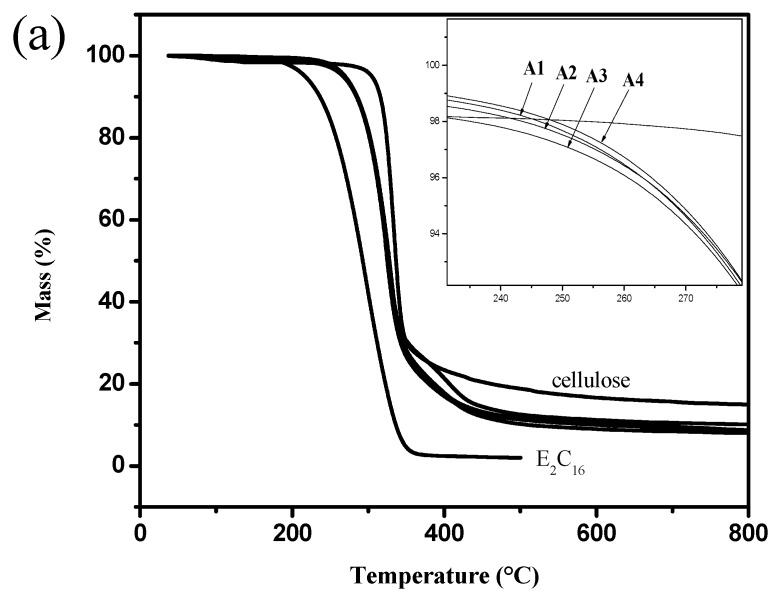

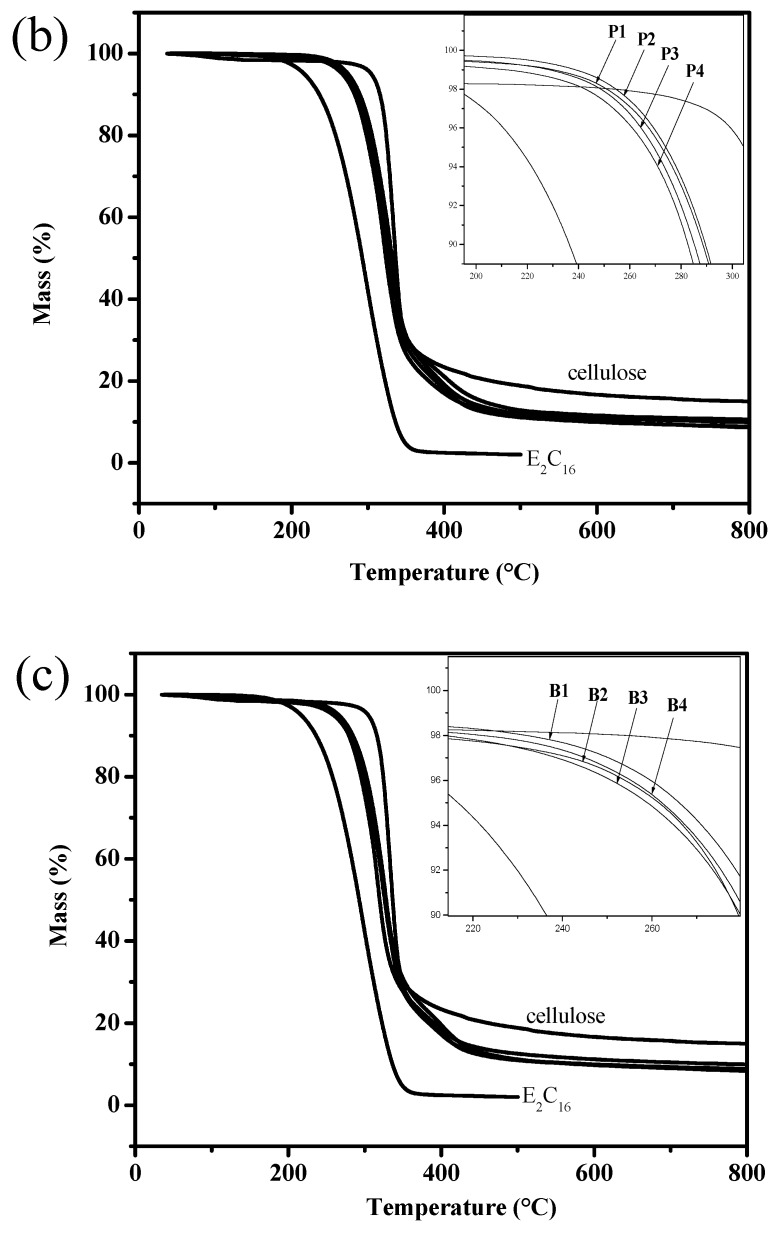
TG analysis of cellulose, E_2_C_16_, (**a**) CA-g-E_2_C_16_, (**b**) CP-g-E_2_C_16_, and (**c**) CB-g-E_2_C_16_ copolymers.

The thermal stability of cellulose, E_2_C_16_, CA-g-E_2_C_16_, CP-g-E_2_C_16_, and CB-g-E_2_C_16_ copolymers were investigated by TG. It can be observed from Figure 6 and Table 4 that all the copolymers are not degradable and that almost no mass loss occurs before 280 °C. The onset decomposition of cellulose occurs at 319 °C [16]. The thermal stability of CA-g-E_2_C_16_, CP-g-E_2_C_16_, and CB-g-E_2_C_16_ copolymers dropped to 294 °C, 288 °C, and 289 °C, respectively. The lower thermal stability mainly is a result of the introduction of the fatty acid anhydride and E_2_C_16_, which significantly decrease the crystallinity of cellulose [17]. Furthermore, the presence of grafted side chains on the cellulose backbone was confirmed. It is worthy to note that the T_do_ of CA-g-E_2_C_16_, CP-g-E_2_C_16_, and CB-g-E_2_C_16_ copolymers were still much higher than that of the raw E_2_C_16_ due to the confinement derived from the cellulose backbone, and were almost independent of the DS value. Compared with pure E_2_C_16_, the thermal stabilities of the cellulose-g-E_2_C_16_ copolymers were improved by approximately 42~50 °C. It is also shown that the thermal stability was improved gradually with the increase of DS. It was concluded that CA-g-E_2_C_16_, CP-g-E_2_C_16_, and CB-g-E_2_C_16_ copolymers can be applied as a type of SSPCM and show thermal stability in a high-temperature environment.

### 3.6. WAXD

**Figure 7:**
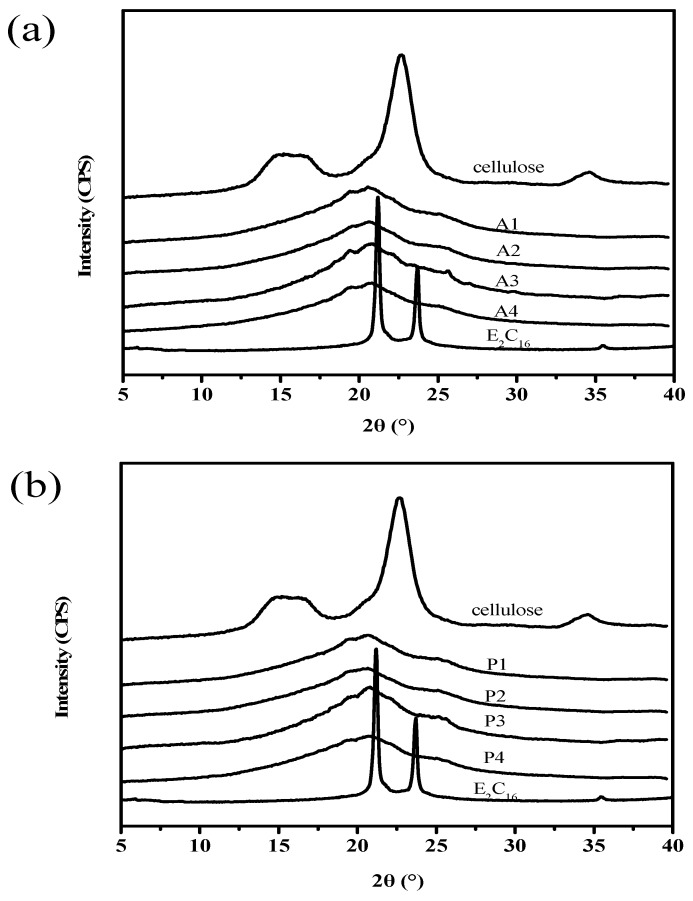

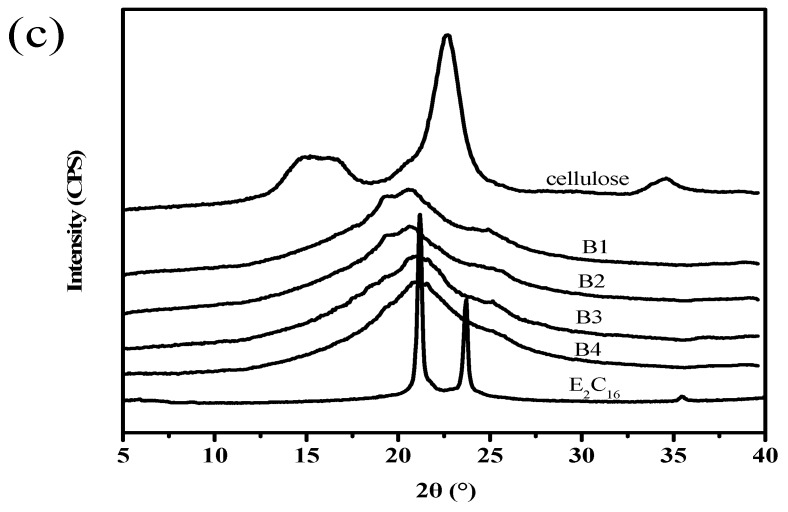
WAXD patterns of cellulose, E_2_C_16_, (**a**) CA-g-E_2_C_16_, (**b**) CP-g-E_2_C_16_, and (**c**) CB-g-E_2_C_16_ copolymers.

**Figure 8:**
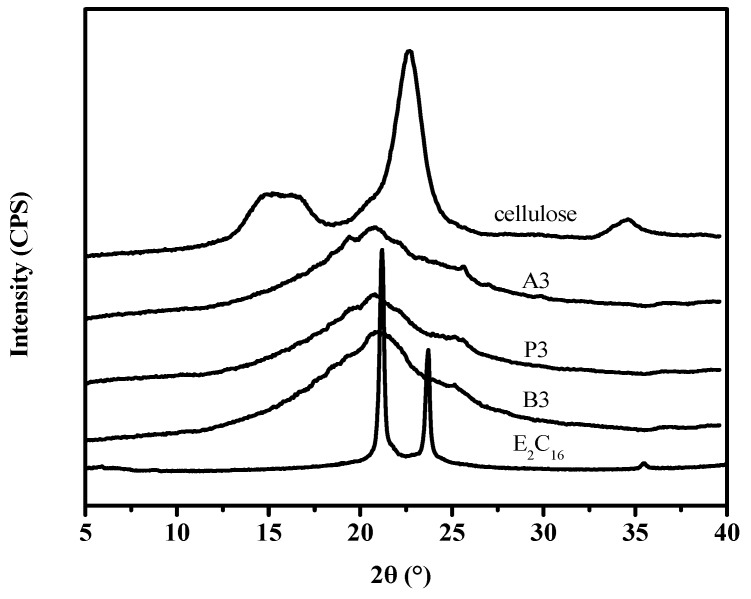
WAXD patterns of cellulose, E_2_C_16_, CA-g-E_2_C_16_ (A3), CP-g-E_2_C_16_ (P3), and CB-g-E_2_C_16_ (B3) copolymers.

Figure 7 shows the WAXD patterns of cellulose, E_2_C_16_, CA-g-E_2_C_16_, CP-g-E_2_C_16_, and CB-g-E_2_C_16_ copolymers that were obtained to investigate the change in the crystal type of E_2_C_16_ before and after grafting. There are three diffraction peaks in the WAXD pattern of cellulose, whose locations are 14.8°, 16.4°, and 22.6°, and the d-spacings are 0.60 nm, 0.54 nm, and 0.39 nm, respectively. E_2_C_16_ shows two sharp peaks centered at 21.2° and a small peak centered at 23.7°, with d-spacings of 0.42 nm and 0.38 nm, and the crystal type of E_2_C_16_ is hexagonal [18]. It is reported that the characteristic diffraction peaks (110) and (020) of cellulose II are locate at 19.9° and 22.1° [19]. It can be seen that CA-g-E_2_C_16_ copolymers show diffraction peaks centered at 20.7° (d-spacing = 0.43 nm) and 21.6° (d-spacing = 0.41 nm), which were close to the characteristic diffraction peaks of cellulose II (2θ = 19.9°) and E_2_C_16_ (2θ = 21.2°), respectively. However, the intensity of the three types of cellulose-based copolymers were all weaker than that of E_2_C_16_ and cellulose itself, as expected. It is demonstrated that the transformation from cellulose I to cellulose II also occurred after the dissolution and regeneration in AmimCl. The analysis above indicated that the crystalline form of the grafted E_2_C_16_ side chain onto the cellulose backbone is not affected by the cellulose molecule. Moreover, CP-g-E_2_C_16_ (P3) and CB-g-E_2_C_16_ (B3) copolymers also showed the same phenomenon. Figure 8 shows contradistinctive WAXD patterns of CA-g-E_2_C_16_, CP-g-E_2_C_16_, and CB-g-E_2_C_16_ copolymers. It can be seen that the diffraction peak locations of these three copolymers are consistent, which indicated that a formed short side chain had no effect on the crystal type of E_2_C_16_.

## 4. Conclusions

Cellulose was esterified using short-chain fatty acid anhydrides (acetic anhydride, propionic anhydride, and butyric anhydride), followed by grafting of polyoxyethylene (2) hexadecyl ether (E_2_C_16_) using toluene-2,4-diisocyanate as a coupling agent and with the feeding molar ratio of E_2_C_16_/AGU fixed at 4:1, and then a series of CA-g-E_2_C_16_, CP-g-E_2_C_16_, and CB-g-E_2_C_16_ copolymers were successfully obtained. It is shown that with the anhydride/AGU ratio increasing, the DS value of E_2_C_16_ was first increased and then decreased. With the carbon chain length increasing, the DS value of E_2_C_16_ showed a trend of increasing continuously. The phase transition temperature and enthalpy of the copolymers increased with increasing DS value of E_2_C_16_. When the ratio of butyric anhydride/AGU was 1.5:1, the DS of E_2_C_16_ was up to the maximum value of 1.02, and the corresponding melting enthalpy and crystallization enthalpy were 32 J/g and 30 J/g, respectively. All copolymers showed solid–solid phase change behaviors. The heat resistant temperature of cellulose-based solid–solid phase change materials is always higher than 270 °C. After the grafting reaction, the crystallinity of E_2_C_16_ is decreased, but the crystal type is still hexagonal.

## Figures and Tables

**Table 1:** Sample number and raw material feeding ratios.

Sample No.	CA:AGU (mol:mol)	CP:AGU (mol:mol)	CB:AGU (mol:mol)	Pyridine:AGU (mol:mol)	E_2_C_16_:AGU (mol:mol)
S-B	-	-	-	2.5:1	4:1
A1	0.5:1	-	-	2.5:1	4:1
A2	1.0:1	-	-	2.5:1	4:1
A3	1.5:1	-	-	2.5:1	4:1
A4	2.0:1	-	-	2.5:1	4:1
P1	-	0.5:1	-	2.5:1	4:1
P2	-	1.0:1	-	2.5:1	4:1
P3	-	1.5:1	-	2.5:1	4:1
P4	-	2.0:1	-	2.5:1	4:1
B1	-	-	0.5:1	2.5:1	4:1
B2	-	-	1.0:1	2.5:1	4:1
B3	-	-	1.5:1	2.5:1	4:1
B4	-	-	2.0:1	2.5:1	4:1

S-B; A, P, and B are just sample numbers. S-B: Cellulose-g-E2C16 copolymer (no acid anhydride added); A: CA-g-E2C16 copolymer; P: CP-g-E2C16 copolymer; B: CB-g-E2C16 copolymer.

**Table 2:** Degree of substitution (DS) values of different samples.

Sample No.	AA:AGU (mol:mol)	PA:AGU (mol:mol)	BA:AGU (mol:mol)	E_2_C_16_:AGU (mol:mol)	*DS*_1_^a^	*DS*_2_^b^
S-B	-	-	-	4:1	-	1.01
A1	0.5:1	-	-	4:1	0.35	0.59
A2	1.0:1	-	-	4:1	0.58	0.75
A3	1.5:1	-	-	4:1	0.75	0.86
A4	2.0:1	-	-	4:1	1.09	0.52
P1	-	0.5:1	-	4:1	0.36	0.62
P2	-	1.0:1	-	4:1	0.55	0.80
P3	-	1.5:1	-	4:1	0.79	0.97
P4	-	2.0:1	-	4:1	1.00	0.88
B1	-	-	0.5:1	4:1	0.35	0.79
B2	-	-	1.0:1	4:1	0.54	0.93
B3	-	-	1.5:1	4:1	0.75	1.02
B4	-	-	2.0:1	4:1	1.04	0.91

^a^: DS value of short-chain fatty acyl group; ^b^: DS value of E_2_C_16_.

**Table 3:** Thermal transitions of E_2_C_16_, cellulose-g-E_2_C_16_ (S-B), CA-g-E_2_C_16_, CP-g-E_2_C_16_, and CB-g-E_2_C_16_ copolymers.

Sample No.	*DS*_1_	*DS*_2_	$W_{E2C16}$^a^ (wt %)	Heating				Cooling		
				T_mo_^b^ (°C)	T_mp_^c^ (°C)	ΔH_m_ (J/g)	ΔH_m_* ^d^ (J/g)	T_co_^e^ (°C)	T_cp_^f^ (°C)	ΔH_c_ (J/g)
E_2_C_16_	-	-	100	27.6	34.4	104	104	29.2	23.9	95
A1	0.35	0.59	41	5.4	10.4	13	43	15.3	-0.6	13
A2	0.58	0.75	44	6.3	16.5	16	46	16.8	2.8	15
A3	0.75	0.86	45	8.0	18.6	17	47	17.6	3.5	20
A4	1.09	0.52	36	4.5	9.8	11	38	15.0	-1.0	12
P1	0.36	0.62	41	2.8	11.7	12	43	12.8	0.7	13
P2	0.55	0.80	44	9.7	18.3	18	46	14.1	2.7	17
P3	0.79	0.97	46	13.7	23.9	27	48	20.8	9.8	28
P4	1.00	0.88	44	13.2	23.2	21	46	19.4	7.2	21
B1	0.35	0.79	45	5.8	14.0	16	46	18.9	3.3	17
B2	0.54	0.93	46	11.0	21.7	24	48	21.4	9.3	24
B3	0.75	1.02	46	14.2	26.1	32	48	23.3	11.1	30
B4	1.04	0.91	43	13.7	25.0	24	45	21.0	9.5	23

^a^: the mass percent of E_2_C_16_ in copolymer; ^b^: the onset temperature of the melting peak; ^c^: the peak temperature of the melting peak; ^d^: the ΔH_m_ of PCMs if in the condition of blending; ^e^: the onset temperature of the crystallization peak; ^f^: the peak temperature of the crystallization peak.

**Table 4:** Thermal stabilities of cellulose, E_2_C_16_, CA-g-E_2_C_16_, CP-g-E_2_C_16_, and CB-g-E_2_C_16_ copolymers.

Sample No.	*DS*_1_	*DS*_2_	T_do_ (°C)	T_dp_ (°C)	Mass Loss (%)
Cellulose	-	-	319	335	85.4
E_2_C_16_	-	-	246	295	98.0
A1	0.35	0.59	294	331	92.0
A2	0.58	0.75	295	328	89.9
A3	0.75	0.86	295	324	91.3
A4	1.09	0.52	296	325	90.8
P1	0.36	0.62	296	333	90.1
P2	0.55	0.80	295	330	89.4
P3	0.79	0.97	294	325	91.4
P4	1.00	0.88	288	321	91.2
B1	0.35	0.79	296	329	91.2
B2	0.54	0.93	289	317	90.1
B3	0.75	1.02	293	328	91.7
B4	1.04	0.91	290	326	91.1
